# A Variational Approach to Scripts

**DOI:** 10.3389/fpsyg.2021.585493

**Published:** 2021-07-20

**Authors:** Mahault Albarracin, Axel Constant, Karl J. Friston, Maxwell James D. Ramstead

**Affiliations:** ^1^Département d’informatique Cognitive, Université du Québec à Montréal, Montreal, QC, Canada; ^2^Division of Social Transcultural Psychiatry, McGill University, Montreal, QC, Canada; ^3^Wellcome Centre for Human Neuroimaging, University College London, London, United Kingdom

**Keywords:** script theory, social scripts, variational free-energy principle, active inference, Bayesian reasoning

## Abstract

This paper proposes a formal reconstruction of the script construct by leveraging the active inference framework, a behavioral modeling framework that casts action, perception, emotions, and attention as processes of (Bayesian or variational) inference. We propose a first principles account of the script construct that integrates its different uses in the behavioral and social sciences. We begin by reviewing the recent literature that uses the script construct. We then examine the main mathematical and computational features of active inference. Finally, we leverage the resources of active inference to offer a formal model of scripts. Our integrative model accounts for the dual nature of scripts (as internal, psychological schema used by agents to make sense of event types and as constitutive behavioral categories that make up the social order) and also for the stronger and weaker conceptions of the construct (which do and do not relate to explicit action sequences, respectively).

## Introduction

How are humans able to navigate social situations? As social agents, we take for granted that we can and do modulate our behavior as a function of what is socially acceptable in certain kinds of situations. In this paper, we are concerned with explaining social behavior that is shaped according to *social scripts*. Social agents must be able to make sense of their social predicaments, identifying event types and reacting to them appropriately, in ways that cohere with the normative standards apt for a given social or cultural group. The script concept helps.

The concept of script is valuable because it explains the (implicit and explicit) forms of social knowledge at play in social actions, and because it allows us to study the interplay between socially constructed norms and aspects of our biologically hard-wired cognition. Scripts showcase the wide variety of interpretive frames and allowable action available in a social niche. The notion of scripts helps us understand that much of what we take to be universal about human behavior is underwritten by culturally specific narratives.

The script construct has a long history spanning several disciplines. Scripts have been applied fruitfully to study human behavior in different fields, from disciplines centered on individual humans minds and their interactions, such as psychology (Ekblom and Gill, 2016), neuroscience (Allain et al., 2007), and artificial intelligence (Abelson, 1981; Tzeng, 2006), to the social sciences, where it has been used influentially in fields like sociology (Goffman, 1999, 2009; Mahardale and Lee, 2013), criminology (Ekblom and Gill, 2016), anthropology (Singleton et al., 2019), and sexology (Metts and Spitzberg, 1996; McCormick, 2010; Wiederman, 2015). Different definitions of scripts abound, with their different focuses. The concept sometimes is used under different names (action schemas, etc.) (Goddard and Wierzbicka, 2004).

The idea that motivates use of the script construct in these scientific approaches is, at its core, dramaturgical (Simpson et al., 1967; Goffman, 1999). According to its proponents, what enables social agents to act in situationally appropriate ways is a shared set of instructions or normative prescriptions for situationally appropriate behavior (Metts and Spitzberg, 1996). The implication of this view is that, in order to act as a cohesive social group in which every agent knows and enacts their role, agents must share a common body of knowledge (i.e., a script) that prescribes situationally appropriate modes of being (Abelson, 1981). This is metaphorically akin to actors sharing a dramaturgical script, hence the name of the construct. Scripts are used in scientific theories to shed light on how internalized psychological models are integrated with externalized social models, by drawing on a pool of common styles of performance and cognition through contextualized acts (e.g., speech acts) and their ensued actions driven by goals (St. Clair, 2008).

There are some issues that stand in the way of an integrated model of scripts across fields.

The first is that the concept gets implemented differently in different theories and disciplines, which throws doubt on our ability to provide a unique definition that can do justice to all the uses of the term in the literature (Abelson, 1981; Ekblom and Gill, 2016). Similarly, different terms can be used to describe very similar phenomena across domains of study, like script or schema, prepended with terms like social, cultural, or cognitive. Some authors have attempted to unify the concept (Schank and Abelson, 1977; Shore, 1998). In our view, these attempts have had a rather limited success, as the varied senses of the term suggest.

In this paper, we focus on two orthogonal distinctions that we suggest structure discussion on scripts in the literature. The first is a split between “internalist” and “externalist” readings of the construct of script. On the internalist conceptions, a script is defined as a cognitive structure that is typically internal to an agent (e.g., encoded in their brain) and that harnesses information about typified behavioral patterns that are appropriate in specific social situations (Abelson, 1981; Waters and Roisman, 2019). On the other, externalist conception, scripts are cast as the basic fabric from which social institutions are crafted. On this conception, a script is a set of highly codified practices, norms, standards, beliefs, linguistics practices, and rules that make up an institution (Heemskerk et al., 2011; Chentsova-Dutton and Ryder, 2020). Some conceptions are not as easy to split between internalist and externalist, but the way in which an agent interacts and reproduces a script does seem to bear elements of this duality nonetheless (Goddard and Wierzbicka, 2004).

The second is a split between the weak and the strong readings of script. The readings differ on how explicitly a script prescribes appropriate courses of action. On the strong reading, a script is a list of explicit instructions for situationally appropriate behavior, either neurally encoded (under the internalist reading) or implicit in conventions maintained by the institution (under the externalist reading). The strong reading dovetails with work in motor control that casts the process of motor control as the execution of a motor representation, which is cast as a list of explicit instructions for action (Tzeng, 2006). The weak reading of the script construct relaxes the assumption that a script prescribes the precise order of events that it entails. A weak script just encodes or harnesses information about the kinds of factors that an agent might encounter in a given situation type (Abelson, 1981).

Besides some early attempts in the field of artificial intelligence (e.g., Schank, 1972), there still is not an integrative formal model that is apt to do justice to all the variegated aspects and uses of the script construct. This makes it difficult to compare and see commonalities between various theories of the script.

The aim of this paper is to formalize the notion of script using the modeling resources of the active inference framework (Friston et al., 2017a). The hope is to shed light on the multifarious uses of the construct of script as it is used in the sciences that study human behavior. Active inference is relevant here because it may provide the key to formulating an integrative script construct. Active inference is an increasingly popular enactive modeling framework that is used to explain the behavioral dynamics of living creatures, i.e., their patterns of action, perception, emotions, attention, etc. (Da Costa et al., 2020; Hesp et al., 2021). Active inference casts all these processes as Bayesian inference processes. Action selection is cast as the Bayesian model selection of a preferred sequence of motor (or autonomic) movements that is informed by the likelihood of sensory observations; while the environment is cast as accumulating the traces of intentional actions left by agents acting together, thereby changing available sensory observation. Sensory perception and active modification of the environment allow active inference to explain how an ecological niche and its denizens become attuned to each other’s statistical structure (Bruineberg et al., 2018; Constant et al., 2019b). Active inference is an interesting candidate framework to develop a principled, computational model of the dual nature of scripts, as internal schema, and as external social order. We will see that active inference can also accommodate both the strong and the weak reading of scripts.

The argumentative structure of this paper is as follows. In the first section, we review the internal, external, weak, and strong readings of the script construct. In the second, we introduce active inference and review the core tenets of the approach. Next, we propose a computational interpretation of the weak and strong, and the internalist and externalist readings of scripts. We show that the modeling resources of active inference can be used to derive a formal construct of script that encompasses the various readings in the literature. We conclude with remarks on the manner in which the proposed active inference model of scripts could be used to further research on human behavior.

## Script Theory: Background

Scripts harness the knowledge involved in situationally appropriate behavior to achieve an intended social goal. Scripts are especially relevant in situations where there is uncertainty concerning the intent of the social partner. The appropriateness of a script is bound to cultural context. Take for instance flirting. The North-American middle class traditional flirting script has been aptly described by Metts and Spitzberg (1996). The flirting script involves signaling one’s intent by incrementally ensuring that similar intent is shared. This entails:

(1) Engaging in discussion on a topic.
(2) Expressing non-verbal behaviors that do not provide evidence that the agent will reach the intended goal (sexual intercourse).
(3) Moving toward topics and behavior related to the intended goal.

Steps 1 to 3 take the form of engaging in small talk; and if it is reciprocated, of choosing to ask more personal questions (e.g., moving from more distant to more personal ones) and enacting behavior (e.g., increasing closeness) that conforms to the interaction goal. If one pursues the flirting script, and starts behaving in a way that is more sexually forward, but their potential partner does not respond or enacts another script, it is safer to assume that they are not interested in engaging in a sexual encounter.

This sequencing says something about the relation between internalization of scripts and the assumption of normalcy or universality. In certain subcultures, like the swinger community (Kimberly, 2016), or gay saunas (Brown et al., 2005; Kimberly, 2016), the reality of the social scripts is equally codified (e.g., with specific locales and ways of acting), but leads to the outcome (sex) differently. Scripts speak to social goals, and communicate the enactment of these shared goals to the people around us.

### Internalism: Scripts in the Behavioral Sciences (Psychology, Neuroscience) and in Artificial Intelligence

The most influential of the social script construct rendition is an internalist one that comes from its use in cognitive and social psychology (Schank and Abelson, 1977; Abelson, 1981). The construct has been used to implement the tacit knowledge that agents have of the social-cultural norms that determine the appropriateness (c.f., prior probability) of behavior in a social situation. Scripts are higher-level constructs that capture fairly general information about how certain tasks are to be accomplished.

The use of scripts by agents can be broken down into two phenomena: competence and performance (Royle, 2013). Competence is the ability of an agent to understand what each social situation entails, what scripts may be enacted, and what are the proper cues indicating when to “enter into” a script; while performance consists in acting on that capacity, leveraging the perceptual and knowledge-based aspects of the script to bring about the situationally appropriate sequence (Ekblom and Gill, 2016). Thus, a script is anchored in a specific social context, and adapts a pattern of actions to the demands of a situation.

Some features of scripts are recurrent in the literature. They must be stable in time, learned from experience and drive behavior (Mahardale and Lee, 2013; Waters and Roisman, 2019). Abelson (1981) nicely summarizes the main features of behavior driven by shared scripts:

*“Three conditions seem necessary for scripted behavior to occur. First, the individual must have a *stable cognitive representation* of the particular script. Second, an *evoking context* for the script must be presented. Third, the individual must *enter the script*. This third is the critical condition at the gap between cognition and behavior. It is assumed that script entry is contingent upon satisfaction of an action rule attached to the script representation.” (Abelson, 1981, p. 791, emphasis added).*

An agent must be able to navigate a social situation, and the psychological script concept gets used to explain the kind of tacit or explicit knowledge at play in the generation of appropriate actions. The first point of Abeldon’s definition says that the “representations” (internal models or schema) of the action sequence to be accomplished must be stable enough to be deployed by the agent in the generation of context-sensitive behavior (Abelson, 1981; Waters et al., 2015).

The second says that agents must be able to recognize situationally specific cues that indicate the appropriateness of enacting a script now (what Abelson calls an “*action trigger*”). Agents must thus be endowed with some knowledge about what environmental cues indicate in terms of appropriate action; and this knowledge is harnessed in the script, which contains an action trigger. Using scripts, the agent may find its way in any situation by understanding which part of the event sequence she is currently in, and how to move forward (Ekblom and Gill, 2016). The enactment of a script following an action trigger assumes an action rule that defines when to partake in the script (Schank and Abelson, 1977; Abelson, 1981). These thresholds may, for instance, comprise *role* definitions. A role is entered and may be replicated in time. Inference about the role of the self, informed by the script, thus guides performance (Schank and Abelson, 1977). Similarly, observation of the actions of another agent can help an agent infer their role in a script, and predict the next actions (Ingelsböck and Schüßler, 2019).

Finally, the last point of this definition says that once the action trigger is recognized by the agent, a sequence of actions to execute must be chosen by the agent. This implies a kind of commitment to the action policy (“entering a script”) by taking on a role in the script. This entails that an agent should be able to perceive possibilities for acting according to the script, and accordingly enact it via performative acts (Mahardale and Lee, 2013).

Modifying the knowledge around the script allows the agent to change their behavior in turn (Abelson, 1981). The agent learns variations of the scripts, and has some part in deciding which way to enact it. Deciding which one to pick depends on the conceptual clusters that can be found in the local environment. These clusters are created directly via analogy or conditioning (Abelson, 1981; Singleton et al., 2019).

Scripts are in this sense similar to narratives (Bouizegarene et al., 2020). Scripts emerge from interactions with relevant social others in situations with which the agent is familiar; and the proximity with narratives comes from the causal relations between events (Ingelsböck and Schüßler, 2019). Scripts harness knowledge related to contexts by specifying possible connections between event types. Scripts harness socially shared assumptions and structure inference that are allowable in a given context (Waters and Roisman, 2019).

In artificial intelligence, the script construct was employed to codify the bodies of regimented inference that are employed by agents in social contexts. The earliest conceptions of social scripts in artificial intelligence consisted in semantic networks structured into goal-oriented sequences (St. Clair, 2008). In this context, scripts were explicit conceptual representations of expected event sequences that were activated by textual triggers. These expected event sequences allow users to bridge gaps in between events because of the logical (e.g., causal) relations encoded in the event sequences (Greenberg et al., 1998; St. Clair, 2008).

In summary, in the behavioral sciences and artificial intelligence, scripts have been used to account for the accurate interaction of an agent with their context. The internalist framework in the social sciences thus focuses on the cognitive schema, permeated by contextual.

### Externalism: Scripts in the Social Sciences (e.g., Sociology, Anthropology, Criminology, and Sexology)

On the alternative, externalist reading of the construct, scripts are related to the existence and maintenance of social institutions (Ingelsböck and Schüßler, 2019). Berger and Luckman compare institutions to theatrical performances, in which actions are programmed and are embodied in a set of specific *roles* that get enacted by social actors (Simpson et al., 1967). It is the enactment of these patterned roles that keeps scripts and the institutions that they compose alive (Turner and Biddle, 1981). Scripts can specify actions for more than one agent; and the various clusters of actions that can be performed by a given agent in a script is called a role.

In the externalist conception, constrain social experience by harnessing institutional norms of allowable behavior (Vanclay and Enticott, 2011). The script construct is here used in a way that emphasizes the social reality that is constituted by the enactment of cohesive, institution-specific modes of acting. Scripts are cast as the building blocks for coherent communities with shared values (Wierzbicka, 2002; Singleton et al., 2019). Scripts are higher level constructs as well on this conception, but these constructs have a social reality outside the mind of the individual.

This poses a problem. Most psychological theories would have scripts exist in the mind of individuals, but this does not explain how they are translated into material reality. By material reality, we refer to the physical properties of the world which carry social meaning. While symbols can take on a material form, not all study of symbol looks at this manifestation, and focuses rather on immaterial properties. We wanted to highlight this second side of the study of symbols. Symbols, materials and culture are intertwined in ways that make them more complex to study separately. Arguably, they can be considered different pieces of the larger social realm. Symbols act as building blocks. They allow meaning to be imbued to units. Materials are often referring to the physical reality individuals have access to, but they can be also associated to the direct ecological context an individual is embedded in. Culture is the matrix which connects and coordinates across individuals the mapping between materials and symbols. The solution starts from noticing that social structures emerge from socially organized psychological phenomena (St. Clair, 2008). The repeated enactment of scripted actions allows agents to make reliable inferences about themselves and other agents. Goffman argued that institutions can be understood as pre-negotiated inferences that find their confirmation in the reifications of practice and language. That these inferences repeat over time helps them crystallize, as it were, into a largely shared common ground (Goffman, 1999; Ingelsböck and Schüßler, 2019). The reiteration of structures does not rob agents of their agency: the agent must take part in the scripts and deal with the unexpected possibilities by interpreting the patterns accurately (Binder, 2007; Ingelsböck and Schüßler, 2019). Scripts, on this conception, are thus overarching structures that contain templates upon which agents can draw for more specific performances (Graesser et al., 1980).

Definitions of scripts in the social sciences differ in terms of the approaches to the study of cultural organization they belong to. We can distinguish between three approaches (St. Clair, 2008): the symbolic approach, the activity theory approach, and the individualistic approach. The symbolic approach (Adler et al., 1997; Ratner, 1999) casts individual agents as the main bearers of power. This power is externalized by the creation and consolidation of shared symbols. This conception accords much importance to shared meanings and concepts, and is far less concerned with the material reality of institutions. Symbols have a life of their own and allow agents to communicate with others and to develop an identity. The symbolic designations of things in the world turns them into mental objects, imbued with meanings and goals (Adler et al., 1997; Ratner, 1999; St. Clair, 2008).

The activity theory approach (Ratner, 1997, 1999) focuses on practice (praxis), and casts psychological phenomena as formed by individuals engaged in social action. This approach, influenced by Marxist theory, emphasizes the material reality of the social world, casting human agency as shaped by the pursuit of meaning and goals in a material social context. Marxist theory casts a specific importance on materialism, and the socially embedded meaning of the material reality. In Marxism, humans search for meaning through the lens of social goals. Activity theory similarly embeds the search for meaning inside the social realm, and places activity and productions as vectors for understanding individuals’ relations to materiality. It is also to note that Leontiev and Vygotsky were both strongly influenced by Marxist materialism, which is perceptible in the theory’s focus on material conditions as social vectors for meaning. This approach studies social phenomena as a function of how power is divided among social agents and how actions are defined by this division of power. Activity theoretic conceptions of the script emphasize individuals’ interactions with the material world. By focusing on a goal, and by being constrained by linguistic tools and practice conventions (i.e., by scripts), humans achieve a stable social order (St. Clair et al., 2005).

The individualistic approach (Garey and Wikan, 1998; Ratner, 1999) maintains a duality between individuals and culture, and proposes that the individual has agency in the way that they objectify culture. Individuals confer meaning that serve their aim to elements of culture, which they will then use to further their aims. Their cognitive life is thus shaped by cultural artifacts, which are objectified pieces of culture that they select for themselves and that they can share with other individuals. Somewhat similar to meme theory (Dawkins, 2016), this approach views culture as fractured quasi-genetic fragments that can be used, transformed and spread (Garey and Wikan, 1998; Ratner, 1999; St. Clair, 2008).

Overall, these approaches to cultural organization in social sciences allow us to portray scripts as a cultural framing. Scripts, as the order social structures, prescribe what an individual should do, given his timestep in a given pre-organized script, and the role they have chosen or was given to embody. This framing is conveyed by the individuals in a group through language and common practices. These approaches emphasize the disconnect between cognitive structures and social practices, which is bridged in language: performative externalized cognition. By performative externalized cognition, we mean that this aspect of cognition is extended to the social realm. Through linguistics, individuals can share and gather information more efficiently, without having to get the information for themselves. The interesting phenomenon at play here is the fact that this social exchange has a weight. Through communicating, individuals create the reality they seek to exchange. Their enactment of this linguistic exchange creates the reality, therefore making it performative. Existence does not happen in a silo, and all thought or action take place within a context. If the context is constituted by relevant social others, language can act as the bridge between self and the social context. This commonality frames what is possible, and anchors what is possible in a specific set of goals (St. Clair, 2008).

### The Strong and Weak Conception of Scripts

#### Strong Scripts

The way the script construct is used in the literature is further complicated by another distinction, introduced by Abelson (1981), between a strong or weak conceptualization of the construct. The general distinction is that the strong concept entails that the script represents events and actions to be performed in a particular order, whereas the weak concept eschews any such ordinality. The strong concept of script has more to do with the links between the concepts present in the script (i.e., their ordering) than the concepts themselves; while the weak concept is more semantic, and speaks to what is typical in a kind of social situation.

A strong script refers to the sequences of structured behavioral events performed by social agents. These can be reorganized by variations, but overall maintain some similarity in structure (Leclerc and Wortley, 2013; Ekblom and Gill, 2016). It is this similarity that enables agents to make inferences about relevant social others (Berg and Hochstetler, 2016). The order of the events is paramount in strong scripts, as they are causally related (Abelson, 1981; Leclerc and Wortley, 2013). Agents are able to infer the next plausible event or course of action based on the temporal and causal connections they know to exist between two categories. The order thus matters because there is a necessary entailment of the social actions. Here, what Abelson called “event triggers” become crucial and act as floodgates, without which the rest of the script cannot or will not be enacted.

Consider for instance ordering food at a restaurant, which is covered in Schank and Abelson’s famous CITE script (Schank and Abelson, 1977). The typical restaurant script in North America is something like the following: (1) Make a reservation or wait in line; (2) Be seated by the host; (3) Review the drinks menu and order drinks; (4) Review the food menu and order food; (5) Eat food; (6) Pay for services (including a 15% tip). Here, order matters. For instance, in most swanky restaurants, it would be considered inappropriate to sit at a table unless first instructed to do so by the host. In European pubs, one typically pays before eating, whereas in American bars, one pays after eating. Failure to conform leads to social friction and might also lead to penalties.

Essentially, the strong sense of scripts can be reduced to a socially coded drive toward goals that also allows agents to infer each other’s goals (Ekblom and Gill, 2016). Because the scripts have common sequences that lead toward a common goal, agents can infer each other’s goals, and infer the next likely actions for themselves, and by other agents. Knowledge is cached in the common practices and the expected goals. Thus, we can consider that a practical aim of scripts and their learning and transmission is to transfer practice-based knowledge. Navigating social interactions is enabled by the reiteration of practices, and optimizes communication between agents. Information is cached in the scripts, and limits how much any given agent needs to learn about optimal existence in his context (Goffman, 1999; Ekblom and Gill, 2016).

#### Weak Scripts

Weak scripts specify the typical features that an agent will encounter in an event type. The order of the events is not specifically important, so much as the semantic proximity and restrictions offered by the boundaries around a concept (what it does and does not contain). In this way, semantic relations are more important than the sequence of causal relations.

Consider for instance the script of going to the library. Some parts of this overall script have a strong aspect: For instance, before leaving the library with a book, one must have withdrawn it from the front desk and registered it under one’s name. However, many aspects of the library script do not depend on an ordinal sequence of events. For instance, it is part of the library script that one should be silent in the library.

Weak scripts are thus, at base, clusters of associations or contingencies related to specific events. Event types do not have to be sequenced ordinally to be semantically connected in this way. In depicting what is typical in a situation type, weak scripts offer a cognitive framing related to contextual goals. They adapt the perceptual field, and make salient possible drives toward actions, and opportunities for roles for the agent (Abelson, 1981; Tzeng, 2006).

Sets of categories have overlapping characteristics which link them semantically (Tzeng, 2006). The more common ground there is between two sets of categories, the more likely two events drawing from these sets are to be in a given sequence. Thus, conceptual proximity defines the overall structure of the weak script and defines a conceptual mapping for given situations. Conceptual mapping refers to the manifold that concepts are made of. Concepts are embedded nested structures of metonymy. They are made of layers of referents, all pointing to hierarchies of smaller or more fundamental ideas. Concepts are essentially webs of lower level semantic units. One overarching concept is the specific configuration of a semantic network, which corresponds to a specific mapping of one idea to many. These mappings for concepts can be different. This proximity structure may also help provide a restructuring effect for the strong sequencing of events in weak scripts (Abelson, 1981).

Weak scripts thus function as semantic markers of sorts, enabling an individual to infer how best to adapt to the demands of a social situation. Specifically, not all variations of a strong script are immediately translatable to a situated context. The weak script allows the agent to find the appropriate action, given the semantic field in which they are engaged. Weak scripts are integrated through practice and help agents imbue situations with meaning. When this meaning is made salient, it can help an individual navigate toward the appropriate and shared social goal (Lydon et al., 1997; Heemskerk et al., 2011).

### Script Variations

As mentioned above, weak and strong scripts evince variety in the way they are integrated and performed. Scripts are polysemous (Greenhill and Fletcher, 2009). One reason for this polysemy is that *several actions that lead to the same result*. There may be more or less typical ways to achieve a given result (Abelson, 1981). Subsequently, some parts of the scripts are more important to the completion of the script, and the events in between these parts can vary. This refers to the gating of strong scripts by action trigger.

Some parts of the script are not as important, and may be at times skipped if the situation allows for it. This allows for several versions of the same overarching script to lead to similar (i.e., the more important) outcomes (Abelson, 1981). Scripts may share similar clusters, or event sequences. These may be linked into tracks or decision trees, which inflect at script gates. Script variations are partly due to the possibility that individual agents show variability in its interpretation and application. However, by considering certain parts of the event sequences to be more important, the possible permutations of scripts are limited. The conceptual “gravity” around certain concepts constrains how one may enact any given action possibility (Schank and Abelson, 1977).

Scripts can also vary because environments vary. The enactment of any given script will not always have the exact same form. There is an abstract nature to scripts that has more to do with the prototypical structure of practice (Ekblom and Gill, 2016). The same sequence may mean different things to different people based on what they previously associated to it. This intrinsic variation can have effects on practice, or it can have effects on whether or not an individual chooses to enact a script in a situation.

This concludes our review and overview of the uses of the script construct in the recent literature. We now turn to the active inference approach, which provides us with the tools to formulate an integrative and formal account of scripts.

## Active Inference: The ABCs

### Introduction to Active Inference

In this section, we leverage the apparatus of the active inference approach to provide a formal model of scripts that is apt to account for all the dimensions of the construct discussed above (internalist, externalist, strong, and weak readings). Active inference is an increasingly popular behavioral modeling framework that descends from older, closely related Bayesian approaches to the brain and behavior, such as predictive coding (Friston, 2010; Friston et al., 2017b). Active inference casts perception, learning, cognition, and action as forms of (Bayesian of variational) inference.

Technically, active inference says that perception, learning, cognition, and action all function to minimize an information theoretic quantity called variational free-energy (Friston et al., 2017a; Friston, 2019). This variational free-energy was first developed in the context of complex statistical inference to finesse intractable inference problems (Feynman, 1972). In this context, we aim as scientists to estimate some unknown probability distribution; however, the computation of such probability distributions often requires marginalizing over an infinite number of states, which makes inference intractable for analytic (exact) procedures. Instead of computing the distribution directly, variational inference allows us to write down a guess about this distribution (the variational or recognition model); variational inference methods to finesse this guess by changing its parameters (i.e., its shape) until it becomes close enough to the target distribution. This closeness is obtained by minimizing a variational (free-energy) bound on the evidence for our (the brain’s) models or hypotheses about how (sensory) data were caused.

Generically, minimizing variational free-energy minimizes the discrepancy between the data that one would expect, given some model of how that data was generated (what is known as a “generative model”), and the data actually obtained (Feynman, 1972; Beal, 2003; Winn and Bishop, 2005). These methods were imported into neuroimaging neuroscience for various imaging modalities (functional magnetic resonance imaging, electroencephalography, and magnetoencephalography) in the 2000s (Friston et al., 2003; Kiebel et al., 2008). When minimized, the variational free energy scores the quality of the model in terms of its evidence, i.e., the probability of those data and that model. In this context, neuroscientists evaluate the probability of different models for how some neuroimaging data was generated. This is called dynamical causal modeling (Friston et al., 2003). In short, the variational free-energy is used to score the probability of each model, given the data; and the model associated with the lowest variational free-energy is the one deemed the best, or most likely to have caused our data.

Active inference applies the same strategy to modeling another kind of data: the sensory data that is generated by the activity of living creatures (Friston, 2019). In this context, the variational free-energy construct is redeployed, now as a measure of the discrepancy between the observations the agent expected to make, and the actual sensory states encountered an agent. Active inference provides a mechanics of belief-driven action: it will look as if organisms select the actions that minimize free-energy (Ramstead et al., 2019). That is, according to the active inference approach, the dynamics (i.e., the behavior) of living systems serves to garner evidence for their existence as agents (Hohwy, 2016). Successful action in the environment generates sensory consequences that are consistent with our preferences; active inference formalizes this idea.

**FIGURE 1:**
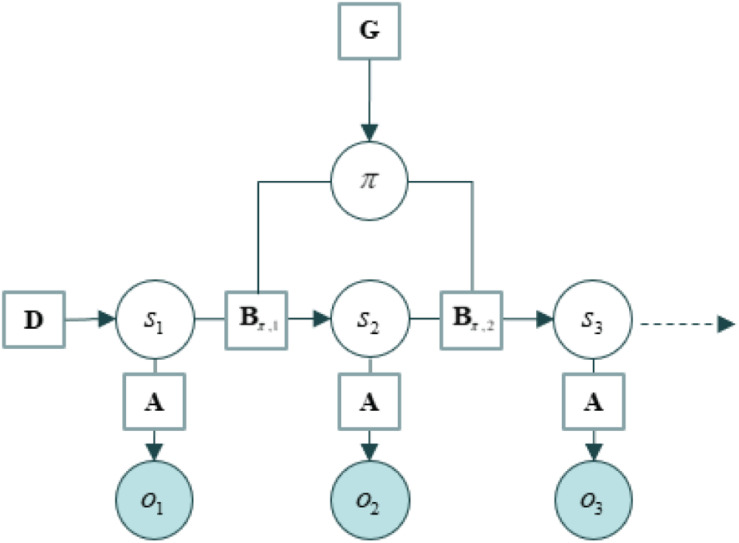
A simple generative model for policy selection. This schematic depicts a generative model for policy selection. It represents probabilistic beliefs about how observations are related to the states that cause them (the likelihood matrix, which is denoted A), beliefs about the manner in which states of the world evolve over time (the state transition matrix, denoted B), and beliefs about states prior to sampling the world (prior beliefs, denoted D). Preferences over outcomes (C) are not depicted. From Friston et al. (2017b). For ease of visualization, we do not present the hierarchical structure of the generative process. The reader should assume that there structure of the generative process will include multiple levels. The important aspect of this schematic is to present the manner in which the generative model and the generative process interact with one another. The only reason we present multiple levels of the generative model is that two levels allows for a description of all the inner components of the script. Only one level of the generative process is needed to describe the external component (even though we should assume multiple levels of the generative process). The higher levels of a model constrain possible inference at the lower levels by unfolding over slower timescales and by setting the prior beliefs about initial states D at the lower level–that contextualize the ensuing state transitions or narratives. In such models, posterior estimates of successive states at the lower level become data or observations for inference at the level above.

These expectations are harnessed in generative models, which do most of the heavy lifting in the active inference approach. A simple generative model is shown in Figure 1 below. This generative model harnesses a number of different probabilistic beliefs: beliefs about the likelihood of observations, given the states that cause them (which are denoted A), prior beliefs about the manner in which states of the world evolve over time (denoted B), prior preferences over outcomes (C), and beliefs about states before sampling the world (denoted D).

A *generative model* is a statistical model of the causal process that generated the sensory data (the aptly named *generative process*). Model and process are linked at two points: the data itself, which the world generates; and the actions selected by the agent, which leave traces in the world and produce typical sensory consequences. Indeed, the main function of generative models is to mediate *policy selection* (or the selection of actions). This takes a particular form in active inference; namely, as the *realization of beliefs about action*. Policy selection is implemented as the selection of beliefs about state transitions, which reflect knowledge about the consequences of action (i.e., as the selection among a series of B matrices, each entailing a different plan of action or path into the future). The state transition beliefs are constructed to incorporate beliefs about the consequences of action; and an action is a series of such beliefs. Active inference gets its name from treating action selection as a form inference about what I must be up to: on the assumption that what I am doing minimizes variational free-energy, given my beliefs about what I might be doing and given my sensory (especially proprioceptive) data, what must I be doing? In some circles, this is akin to “planning as inference” (Attias, 2003; Botvinick and Toussaint, 2012; Millidge, 2019).

Importantly, in many applications, the generative models have a hierarchical or deep structure. Typically, the higher levels of a model constrain possible inference at the lower levels by unfolding over slower timescales and by setting the prior beliefs about initial states D at the lower level–that contextualize the ensuing state transitions or narratives. In such models, posterior estimates of successive states at the lower level become data or observations for inference at the level above.

In active inference, goals are not specified in terms of preferred states, but rather in terms of a preference distribution over outcomes (which is denoted C); that is, in terms of the preferred consequences of action. Motor control is then based not on the computation of explicit motor commands, as in optimal control theoretic formulations, but instead on feedback, in the form of prediction errors (Friston, 2011). This nicely avoids having to compute explicit motor commands in an intrinsic frame of reference (in terms of states of motor effectors, e.g., in terms of the stretching and contracting of muscle fibers); for a discussion of the implications of this for control theory, see Baltieri and Buckley (2018); Hipolito et al. (2020). In other words, the goal of an action is specified in terms of the preferred sensory consequences of action, rather than in terms of preferred states, and policies are selected that lead to these outcomes. Technically, the C vector enters into the calculation of the expected free-energy G for every policy, and defines the preferred outcomes against which actual outcomes are compared in the computation of the model evidence (negative variational-free energy).

### Shared Generative Models and Sociocultural Dynamics

The active inference framework has been used to explain the emergence of coordinated group behavior. It was first shown that target patterns (e.g., morphologies) can emerge from the group behavior of components individually engaging in active inference–on the condition that all free energy minimizing correspondents share the same generative model, that is, the same model structure with the same parameters (the A, B, C, and D matrices, etc.) (Friston and Frith, 2015; Palacios et al., 2019). When applied to large scale ensembles, such as cultural human ensembles, the convergence of behavior based on the sharing of similar enough generative models is thought to be mediated by the structure of the environment (Bruineberg et al., 2018), such as the ways in which culture is materially implemented (Constant et al., 2018). In this setting, the variational free energy minimum of the ensemble coincides with the corresponding minimum of each constituent. Because the environment is constituted by other constituents like “me” the environment and all of its denizens become mutually predictable.

On this view, a cultural or social group is a group of agents that has some common ground of shared cultural traits by virtue of sharing the same beliefs or expectations about the sensory consequences of allowable, situationally appropriate behavior. Agents acquire this body of common knowledge by the structure and parameters of their shared generative model, based on available sensory observations generated by the causal structure and processes of the environment (Constant et al., 2019a). To account for variations in culture, one needs (among other things) to account for variations in state transition narratives, leading to differentially parameterized generative models, and thus to differentially enacted policies (i.e., inference of a course of action based on the generative model). Variations in outcome sequences, in turn, result from environment-modifying actions, of both the explicit and implicit varieties (as in designing a park and leaving footprints in the snow, respectively).

**FIGURE 2:**
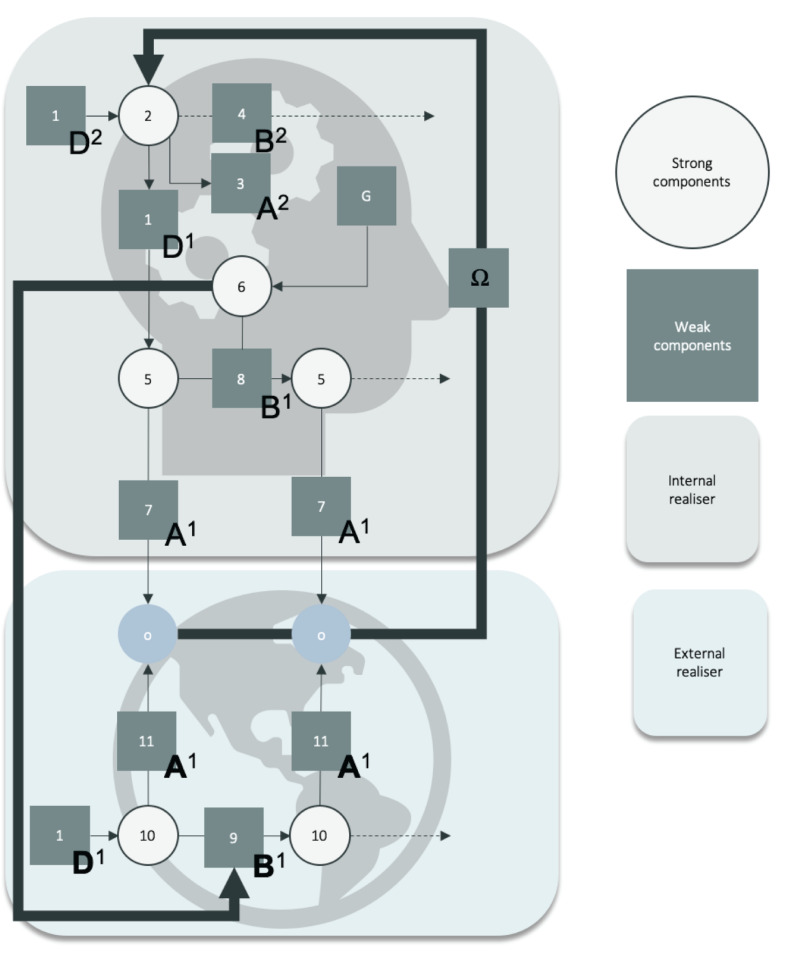
Heuristic description of the generative model of the niche and of the agent. This schematic should be read as a heuristic “formal flowchart” of the biasing relation between priors and likelihood in generative model, rather than as a standard (probabilistic graphical) generative model. Weak scripts correspond to the knowledge about event types and their relation to sensory data available to the agent. Computationally, these correspond to priors and likelihoods (denoted as squares 1, 3, 4, 8, 7, 11, 9) that are combined to infer sequences of hidden states and the action policy (denoted as open circles 2, 5, 6, 10). Note that, for instance, “D1” in the bottom portion of the schematic is not the same as “D1” in the top section since the former is an attribute of the generative process, and the latter is an attribute of the generative model. We use the notion “D1” in both cases to help the unfamiliar reader to visualize the mirroring relation between the generative process and model.

Crucially for our purposes, recent work has suggested that generative models can be constructed with priors that dictate immediately which course of action to pursue upon sensing certain specific outcomes. Technically, the parameter allowing for such precisely inferred, habitised, trigger-based behavior involves a likelihood mapping between observation and policies, and a prior belief over policies forming what is known as “deontic value” (Constant et al., 2019b)” Deontic value is an attribute of the posterior of a policy. The likelihood (A) and priors (the B, C, and D) can be learned based on observations. Taken together, the standard inferential “ABCD” and deontic pathway to policy selection allows us to talk about *policy selection* in the context of cultural *group dynamics* that form via the exchange of local specific sequences of sensory observations on the basis of which a shared generative model can be learned that underwrites–and is underwritten by–a shared exchange with a common econiche. See Figure 2.

In Figure 2, Sequences of hidden states correspond to strong scripts. Action triggers (deontic cues) are represented by filled circles (blue). The ensuing architecture is defined over both internal (head icon) and external (planet icon) states. Weak and strong scripts are realized across multiple levels that span internal and external factors. From 1 to 10: (1) is the prior belief about initial state (e.g., in the flirting script, it is the categorical assumptions about the world, or specifically, the belief that there are men and women in the world, and that they will probably present themselves differently. It is combined with the likelihood (3) at that same level to infer the latent state (2). Prior beliefs about transitions at the second level (4) will contribute to determining what will be the future latent state at the second level. Here, we represent only one cycle of inference at the second level. One cycle of inference at the second level involves two (or hypothetically more) cycles of inference at first level. The inference of the latent state (2) biases the inference of the first state and subsequent states (5 s) (e.g., in the flirting script, the second level entails the belief that an agent is enacting an attractive role, being sexually interesting. They assume that their first state will be to engage the flirting with a low level of intimacy) and the action policy (6) (e.g., you should increase intimacy level with each transition, but you should start at a low level so as not to be unattractive) at level one, which themselves combine a likelihood at level one (7) (e.g., that a given event is mapped to a low level of intimacy, such as saying hello or asking about each other’s name). On the side of the external world, there is only one process in play, which includes the likelihood of observation and states of the world (11) and the transition between these states (9). The agent can act on the transitions (downward thick arrow) to change the sort of outcomes generated by the world. Crucially, the outcomes (o)–that mediate the interaction between world and mind–function as deontic cues, to trigger certain plans or policies, formalized as a prior (denoted omega) biasing the inference about the first hidden state at level two of the agent’s model. These outcomes form a likelihood between sensory outcomes and higher-level hidden states (e.g., the agent can thus check whether or not they have flirted accurately, based on whether or not their observation confirms that they are still attractive to their partner, and change or maintain their behavior accordingly. For instance, if the agent observes that their partner does not reciprocate their questions, and their body language does not increase in intimate proximity, the agent may infer at the second level that the interaction is not attractive and possibly not leading to sex). We also add expected free-energy (the G matrix) over the policy (6) at level one, which includes prior preferences (the C matrix).

## An Active Inference Accounts of Scripts

We leverage the dual aspect of active inference (i.e., its appeal to dynamics internal and external to the agent) to dispel the tension and apparent contradiction between internalist and externalist renditions of the script construct. Active inference can accommodate all the dimensions of the script construct discussed above (i.e., the distinction between strong and weak modalities and the externalist versus internist conceptions) in a way that is both systematic and principled. Active inference can be used just as well to account for the structure and function of externally realized cognitive functions [e.g., extended cognition (Clark and Chalmers, 2010; Chalmers, 2019; Constant et al., 2019a)], as it can be used to describe the internal dynamics of agents; and it provides the requisite flexibility to accommodate the representation of both explicit scripted sequences of events (strong scripts) and typical event type features (weak scripts). We organize the next section as follows: we will examine the strong and weak conceptions of script under active inference, and for each, show how externalist and internalist readings can be accommodated.

### Scripts as Shared Conceptual Structures About Event-Types

The most complicated aspect of the script construct to implement under active inference pertains to the weak conceptualization of the scripts. By weak scripts, we refer to semantic connections between event type concepts. Some concepts are more closely related than others, creating clusters. Some social goals are constituted as clusters. By connecting concepts more or less closely, conceptual clusters offer a cognitive framing related to contextual goals. The weak scripts are just a manifold of unordered semantics webs. They are less formal, and less easily implementable than direct strong scripts, which have a behavioral and measurable component. For example, if we come back to the earlier example of flirting, the goal of having sex entails a variety of conceptual connections. The feelings of attraction, connection, and mutuality are all connected to the flirting script. These categories can also be broken down into more concrete associations. Attraction can be connected to appearance and personality, which are related to physical attributes or behavioral traits.

Through this process of association, an agent can map observations to latent conceptual categories, which crucially include the kinds of things that one typically does in certain types of situations. Once the associations are learned, the agent has adapted their perceptual field, and increases the salience of possible drives toward action and opportunities. By observing salience cues in the environment, the agent is driven to enact specific roles. Hence, weak scripts are closely related to direct perception and conceptual event type structures.

A conceptual mapping is a statistical probability matrix over certain sub-concepts. The manifold that forms the larger concept is a series of probabilities over other concepts. This follows the prototype theory, where some sub-concepts are more prevalent a concept, and others less so. This statistical mapping can be different across groups, and create communication breakdowns. But a niche will share the same probabilistic matrix for a given concept, making coordination easier. By defining conceptual mappings, weak scripts define the probability of certain categories being connected to certain observations, while others are not. Consequently, weak scripts increase or decrease the prior probability that certain kinds of states will be involved in a given event type. Because this mapping is shared by the niche (and indeed, might even be encoded in the physical structure of the environment), the agent can make sense of its environment, and pick up social cues from the niche. Cognition is offloaded to the niche since it reliably furnishes those statistical contingencies over time. The agent has only to pick up and interpret the cues, and does not have to try to figure out the connections between all the possible categories.

From the point of view of active inference, adopting a script allows an agent to minimize its free-energy both by enabling them to avoid spending limited resources sampling elements of the environment at random to figure out which social goal to conform to, and also by limiting the occurrence of errors when trying to achieve that social goal. Technically, scripts play the role of empirical priors that, in effect, simplify belief updating by constraining the degrees of freedom used in modeling exchanges with the (usually prosocial) econiche. Mathematically, this enables an accurate prediction of sensory exchange with minimal complexity, which precludes overfitting. In this setting, complexity is the difference between posterior and prior beliefs, i.e., the degree of belief updating incurred by observing a particular outcome. One can see that if the degrees of freedom of this belief updating are constrained by the right kind of scripts or priors, then there is less latitude for belief updating and a more efficient minimization of variational free energy. A related study by Wirkuttis and Tani (2021) has explored a similar space, related to dyadic interactions governed by active inference surprizal reduction. In their design, they had two robots interact by imitating each other, using active inference. By giving different complexity terms to the robots (tighter and softer) which in turn leads to different agency, the robots will begin to imitate accurately, and thus coordinate. Without such terms, the robots will ignore each other. This dyadic structure is interesting in the specific case of gender as it hints at hierarchical expectations. We expand on their model by offering that surprizal is already limited by existing priors contained in the niche and integrated by the actors.

The conceptual mappings at play in scripts, like those that figure in the generative models of active inference, are probabilistic. Many mappings are shared through narrative construction and practice in a niche, but the agent has a part in the interpretation of those clusters. Variations in the weak scripts of individuals occur, even when they exist in the same niche. Alternatively, individuals from different niches are exposed to different narratives, and hence adopt different conceptual mappings. This explains why some agents, adopting different scripts, do not perceive the same affordances or possibilities for action.

With this in place, it becomes possible to implement weak scripts in a generative model. We submit that weak scripts can be implemented via the likelihood mappings (A), prior beliefs (D), and sensory preferences (C) of the agent. Thus, weak scripts harness beliefs about how the expected salient social categories figure in specific situations (D) and beliefs about how they generate sensory data (A). In social situations, the relevant social categories of role and appropriate behavior can only be inferred, which requires the agent to mobilize the right kind of knowledge. An agent must infer the proper categories, the proper associations, and the proper mapping onto observations in order to navigate a social context adequately and to maximize her returns by the niche (social capital). This mapping changes in function of the context. Hence the weak script also feeds one’s understanding of the context *per se*.

So far, we have only addressed the internalized aspect of the weak script. But the weak script can also be externalized and thereby provides the individual with an ecology of cues that direct their behavior in situationally appropriate ways. The niche has a double aspect: it both is the generative process that causes the agent’s sensory states, and has its own generative model of the social script, physically and discursively offering patterned observations to the agent. The agent measures this against the niche and its model (whether or not other individuals share a specific mapping). For instance, in the flirting script, an agent will interact with another agent. The agent will present themselves physically to signal to other agents that they conform to some norms around attractivity, and that they are interested in a specific type of agent. Agents in the niche must be able to read these signals, and possibly also conform to those norms in order to be recognizable as possible mates by the first agent. If an individual fails to act in a way that is recognizable (i.e., inferable) by other agents, they will not achieve their goal. Other agents will not be able to map appropriate cues on their behavior, and they will either be rejected, or have to update their model, and adapt to the current context.

The niche, with its own external generative model of its denizens, produces observations, patterned in a specific way so as to be reliably interpreted by the agents of the niche. For instance, in the flirting script, showing a positive response to attempts signals that there is a higher likelihood of the flirting being mutual. On a larger scale, a bar offering free drinks to women signals that it promotes a probable heterosexual script of seduction, and that individuals presenting themselves at the bar will probably have to conform to a binary gender frame in order to be legible by one another. By enacting certain social cues that are legible by each other, agents send deontic cues to the other members of the group.

### Scripts as Representations of Typified Sequence of Events

We have considered scripts as referring to clusters of categories that map onto the world, and overlap. They also refer to sequences of actions. This was previously associated with the strong sense of scripts. These sequences are causally related, which means that they are not simply habitual and dependent on practice, but that they also enable agents to make inferences based on knowledge of relations between social categories, which might be interpreted as the hidden factors of a generative model. An agent faced with having to perform the next step in a behavioral sequence will not be at a loss about what to do next precisely because they can infer the causal order of events. The information contained in the strong scripts promotes the possibility for variations, since their causal structure is not deterministic or rote, but probabilistic and open to variation.

Sequencing of the events in the scripts entails a progression over time–a narrative. The direction of this progression is prescribed to the agent by a social goal that the agent must achieve and by the allowable causal order of events. Hence, an individual must not only be in the proper state at a given time–from the inferences drawn from the observations–they must expect and enact suitable transitions as well. Agents must have a model of how events unfold under normal circumstances, but they must also be able to act accordingly, and bring about that state to move to the next causality link, in order to reach for the goal.

We can map these causal sequences in the beliefs about state transitions (B matrices) and preferences over sensory outcomes (C). Social goals are represented in terms of their typical sensory consequences, which are accumulated in the C matrix. Agents have a model of the likely transitions between states, given by the goal state. Sharing beliefs about transitions between states makes the behavior of a social agent more predictable by other agents. In response, the niche’s actual transition probabilities drive the expected social responses to an agent’s actions, which can be considered deontic cues. The agent must not simply predict the next state, they must also act to manifest those states. By following each other’s expected scripts, agents send a signal to one another.

What Abelson called the “evoking context” or “action trigger” pertains to the strong conception of script as well, and might be implemented in active inference as *deontic cues*. The agent receives a cue from the environment as to what action will allow it to achieve its preferred state by increasing the probability of a specific, contextually appropriate policy. Hence, an agent will scaffold policy selection based on prompting and reinforcement from the deontic cues of the environment.

This allows us to understand how a niche can predict a certain pattern of behavior, and strive to provide only the relevant and salient tools. For instance, a bar or pub might provide women with free alcohol, because seduction patterns tend to happen in proximity with alcohol and pubs. The availability of alcohol for women at no cost signals to people interested in flirting with women that some will be there. It also assumes that the only customers will not be women, or else the bar would operate at significant losses; thus, the script incorporates gendered roles. And finally, it assumes that women are less likely to be motivated to enter in such places and must be motivated by an external factor, whereas men will be motivated simply by the presence of women.

This seemingly benign action has many underlying assumptions, which end up portraying two very different roles in the same flirting-at-a-pub scripts for men and women, both in a strong and weak reading. The weak modality of the script pertains to the categories (hidden factors) present in the narratives surrounding the pub context. The strong aspect of the script is suggested in the order of actions drawn from the narrative relationships between the categories, as discussed in the introduction. If the women are portrayed as less motivated, it follows that they are not expected to act overtly or take a leading role in the flirting script.

In scripted behavior, individuals can be making inferences on distinct hierarchical levels. At the first level, individuals infer categorical states from direct observations in their social environment. This state inference is used as an observation for the second level, at which they infer a role being enacted, conferring some stability in the script. The agent has to infer the most likely transition between states at the first level, which will correspond to the next likely event in a social interaction, translated in category states. At the second level, the next likely state corresponds to the continuation of the previously inferred role, or its discontinuation.

The niche both allows the individual to infer the probability of initial states by offering contextual cues, but will also offer feedback to the individual both on whether the role is enacted properly, and what the proper policy to adopt is, in order to maintain the social script.

The niche also plays a direct role. Providing condoms in bathrooms makes the usual goal of seduction very salient. Playing smoother songs by the end of the night responds to the order of the social script of seduction, where individuals will probably end their night together (as opposed to starting directly with sex, and coming back to the bar for post-coital drinks). Dimmed lights and slow music may act as deontic cues for the agents to know they are expected to have reached a certain point in the flirting script. This can be considered by the agent as a deontic cue. By responding to the scripts, the niche constrains the social possibilities of the individual by making salient certain category clusters, and promoting a sequence that, when broken, has stronger social consequences, and is made more obvious for the agent. The environment provides thus patterned regimes of attention to guide the actions of the agent.

## Discussion

Our model has some key differences with previously existing models, such as Gagnon and Simon’s sexual script construct, or Abelson and Schank’s script construct, or even Minsky’s frames. Gagnon and Simon’s sexual script theory addresses the multiple scales at which scripts are enacted, which is in line with out model of scripts based on active inference. Although our present account of scripts entails a model with only two layers, it can be scaled up to become more granular, such as to encompass different time scales, and to encompass varied cultural packages. The beauty of our model is that these notions of cultural structure are no longer blurry. They can be formally deciphered and differentiated in terms of the active inference formalism, and we can study their effects over causal chains.

Another relevant difference is that we accommodate the two main dimensions of the script concept, which no other proposed construct has done heretofore. Sexual scripts are fully internal, according to Gagnon and Simon. While cultural patterns and practices act as scaffolds that enable the internalization of sexual scripts, these are fully integrated in the individual’s psyche. Our model entails no such internal segregation. On our account, scripts enacted in the shared environment and the material conditions of the world fully participate in every scripted situation. Finally, while the scripts of Gagnon and Simon are restricted to the sexual realm, our model can apply to any social situation.

Abelson and Schank’s model, on the other hand, is much more generally applicable. However, it mostly applies to the strong scripts, as it specifically concerns the typified sequence of events that an individual comes to expect in a given situation type. Such sequence-emphasizing theories also include schema theories, such as the gender schema theories proposed by Boston and Levy (1991), Levy and Boston (1994), which posit that individuals learn early on what kinds of behavioral sequences are expected of them based on their assigned gender. This schema theory, however, extends beyond the scripts concept, and describes psychological attributes, such as attitudes and preferences, which cannot be collapsed into scripts. While these attitudes and preferences can be scripted, they extend outside of the scope of the scripts theory *per se*. Our account of scripts does not solely focus on sequencing, however, it also does not reach out of the symbolic space that scripts occupy in the psyche.

On the other end of the spectrum, Minsky’s (1975) frame theory and Bartlett and Bartlett’s (1995) schema theory, followed by McClelland and Rumelhart’s (1985) schematas, are more focused on the weak interpretation of scripts, as they offers a way that we reconstruct incomplete information to paint a picture and assign meaning to a given context. Minsky’s frames are slightly more rigid than our conception of weak scripts in active inference. Specifically, knowledge needs to be relatively certain and re-applicable in generic ways (Minsky, 1975). Our conception of scripts instead relies on statistical probabilities. There is no need for knowledge to be classified in particular categories in order to be semantically linked. Furthermore, these theories can only account for already-known information and do not allow for an account of how new concepts are internalized. Our active inference script theory gives us an account of how knowledge about the world can be updated as the individual encounters new social dynamics. A future account will lay out more clearly how new information is learned in the weak scripts. Overall, what we can see is that our new conception can account for the various parts that the previous accounts worked through separately.

Our proposed formalization of the script construct–via active inference–allows for interesting avenues in social computing. Specifically, we can begin to make predictions about how humans react to scripts. By clearly identifying the formal role of internal and external script elements–as well as what weak scripts and strong scripts entail in a cognitive and ecological structure–we can begin to leverage the model to identify the moment-to-moment dynamics of interactions between social agents in a given context. We can identify how narratives influence expected behavior and contextual framing.

Scripts have generally been used as a framework to aid conceptual analysis. For instance, weak scripts can be used for in historical analysis to assess contextual relevance (Fleer and Robbins, 2004), or to analyze codes in literature and art under the angle of discourse (Tagg, 1992; Sun et al., 2016). Scripts allow us to frame concepts in the context of their larger associative networks to predict whether a concept will be negatively or positively framed, based on shared cultural models and narratives (Miyamoto and Ma, 2011). They have also been used to allow behavioral predictions and motivations in larger scale events, like criminology (Miyamoto and Ma, 2011; Ekblom and Gill, 2016), or epidemiology (Trostle, 2005).

Understanding cultures in those areas allows researchers to make predictions about normative sequences and the consequences of violations of shared norms. However, these models usually take a very abstract, heuristic approach to scripts, and mostly use the script construct as a template to guide analysis, rather than as a relevant prediction tool.

In criminology, the concept of script most often refers to patterns of scripted behaviors (Hayward and Young, 2004; Beauregard et al., 2007). These scripts can have an ecological dimension, but they mostly refer to the strong understanding of scripts. In our model, such scripts could be more accurately predicted, leading to preventive actions and risk limitation. But given our model’s connection to weak scripts as well, working with offenders to undo thought patterns that may support such strong scripts would be made easier.

Similarly, epidemiology uses scripts theory to predict the way individual and group dynamics will influence the spread of a disease, or a problematic factor (Gibbs, 2001; Turchik et al., 2009). As we have seen with the COVID pandemic, such scripts can become vitally important. Our model can offer precise predictions as to the adoption of behaviors, and concepts for different populations, and allow us to predict what kinds of behaviors may be adopted.

For the sexological use of scripts, the ramifications of our model can have deep impacts in the way we can approach patients in therapy. Interpersonal scripts, perceptions and patterns are influenced by the ecology of the individual, and can be rearranged. Keeping these scripts in mind, erotic patterns can be woven and unwoven as they need to be for different types of pathologies.

With our formal model of scripts, we can map the direct interaction of an individual with social categories and events, as well as its concomitants in the shared niche. Those dynamics could allow not only to model how scripts come to be widespread–by simulating sensitivity to deontic cues and social coordination with interlocutors–but also how scripts may come to change when an agent is faced with a script violation, and its different ramifications. These violations may be met with social punishment, or be embraced when they tap into previously invisible, valuable social reality. This formalism may be scaled up to simulate agents as the niche, and see how certain patterns of interactions and co-option may emerge. Finally, the model and the predicted patterns may be measured against real empirical data, and falsified or confirmed to test psychological hypotheses about adhere to scripts.

An interesting avenue, which we are now pursuing, is to test these models against the constructs used in the gender and sexuality studies, which have already used the concept of scripts with its interpretation from Simon and Gagnon (1984). Our formal treatment of sexual and gendered scripts may shed new light on this work and may allow real progress in the clinical and theoretical fields of sexology. Gender studies, rooted in feminist thought, may find new ways to critically address more biological explanations of gendered interactions and gendered differences. This is also an interesting avenue to deconstruct assumptions of the duality between the influences of nature and nurture on development, bringing human sciences into the neo-materialist era.

Future work will be concerned with formalizing the mathematical model and applying this model to simulations. These simulations could then be tested against real observed behavioral data. We can infer from these behavioral data the potential conceptual mapping that was integrated by an individual. We can also choose the alternative route of starting from a conceptual mapping, and deriving behavior, which we can then test against real data as well.

Our conceptualization of script theory accounts for different structures of information, and thus accounts for the manner in which agents flexibly adapt to new situations, learn, grow, and work out uncertainty in their script.

This paper represents the first in a multi-step process, whereby formal models are constructed. The first step is to formalize some informal notions to provide a theoretical account. We now present some speculation about what novel predictions this framework should yield. Following steps include implementing the model, at which point it becomes possible to make quantitative predictions. We can thus expect that our model will allow us to predict the ways in which information on the weak continuum maps directly to sequences, by having these representations modeled as the A matrix for the weak script, and the sequencing as the B matrix. As our model will enable an agent to decide between changing their model of the world and changing their actions, scripts will be able to evolve and integrate new information. The future directions of this research will allow us to explore how entirely new concepts get integrated as concepts. We believe that this will be related to the extent that pre-existing concepts or sub-symbolic concepts get clustered together. With these tools in hand, we should be able to predict social dynamics from large scale groups to interpersonal interactions. We will be able to untangle the ways in which an individual may be embedded in their surrounding causing some pathology and we can highlight the exact paths that lead to systemic and symbolic inequalities.

One important benefit of our model, beyond its precision, may be its computational advantages. Specifically, a lot of complexity can be smoothed out as constants at different levels of state space, which correspond to different rates of change. The interpersonal level will go much faster than the cultural level, and this cultural level, at the scale of interpersonal relationships, can essentially be cast as a prior.

## Conclusion

The aim of this paper was to propose a formalization of the script construct using the apparatus of active inference. We hoped to propose an integrative account of the script construct that does justice to its sundry uses in the sciences that study individual and collective human behavior. First, we reviewed the recent literature that uses scripts. Then, we examined the active inference approach, a behavioral modeling framework that casts action, perception, emotions, and attention as processes of (Bayesian or variational) inference. We then leveraged active inference to provide a principled, computational model of scripts that accounted for the dual nature of scripts as internal schema and as external social order, and for the stronger and weaker conceptions of the construct (which do and do not relate to explicit action sequences, respectively).

## Data Availability Statement

The original contributions presented in the study are included in the article/supplementary material, further inquiries can be directed to the corresponding author/s.

## Author Contributions

MA focused on social scripts theory and its active inference formulation. MR and AC adapted the active inference frameworks in terms of the social scripts theory and produced the figures for the article. KF specialized ensured that our interpretation of the model followed the appropriate mathematical formalism. All authors contributed to the article and approved the submitted version.

## Conflict of Interest

The authors declare that the research was conducted in the absence of any commercial or financial relationships that could be construed as a potential conflict of interest.
